# Impact of the Hydration States of Polymers on Their Hemocompatibility for Medical Applications: A Review

**DOI:** 10.3390/ijms18081422

**Published:** 2017-08-03

**Authors:** Min A. Bag, Loreto M. Valenzuela

**Affiliations:** 1 Chemical and Bioprocess Engineering, Pontificia Universidad Católica de Chile, Santiago 7820436, Chile; mabag@uc.cl; 2 Institute for Biological and Medical Engineering, Schools of Engineering, Medicine and Biological Sciences, Pontificia Universidad Católica de Chile, Santiago 7820436, Chile; 3 Research Center for Nanotechnology and Advanced Materials “CIEN-UC”, Pontificia Universidad Católica de Chile, Santiago 7820436, Chile

**Keywords:** *intermediate* water, *non-freezable* water, *free* water, water structure, platelet adhesion, fibrinogen adsorption

## Abstract

Water has a key role in the functioning of all biological systems, it mediates many biochemical reactions, as well as other biological activities such as material biocompatibility. Water is often considered as an inert solvent, however at the molecular level, it shows different behavior when sorbed onto surfaces like polymeric implants. Three states of water have been recognized: *non-freezable* water, which does not freeze even at −100 °C; *intermediate* water, which freezes below 0 °C; and, *free* water, which freezes at 0 °C like *bulk* water. This review describes the different states of water and the techniques for their identification and quantification, and analyzes their relationship with hemocompatibility in polymer surfaces. *Intermediate* water content higher than 3 wt % is related to better hemocompatibility for poly(ethylene glycol), poly(meth)acrylates, aliphatic carbonyls, and poly(lactic-*co*-glycolic acid) surfaces. Therefore, characterizing water states in addition to water content is key for polymer selection and material design for medical applications.

## 1. Introduction

Water is considered to be the most important compound of life and is the most abundant compound on earth. Over 70% of the surface of the planet is covered by water, in the form of solid, liquid, and vapor [1]. It is also the main component of biological systems, being essential for many chemical reactions. Water is often regarded as the universal solvent because of its great versatility: it can dissolve proteins, ions, sugars, gases, organic liquids, and lipids. What is fascinating about water is its uniqueness. Compared to other common liquids, water is characterized by having high boiling, melting, and critical temperatures, large specific heat, and high surface tension, among other properties [2]. In the first section of the review, general aspects of water and its behavior in biological systems and surfaces are described.

### 1.1. General Aspects of Water

**Figure 1:**
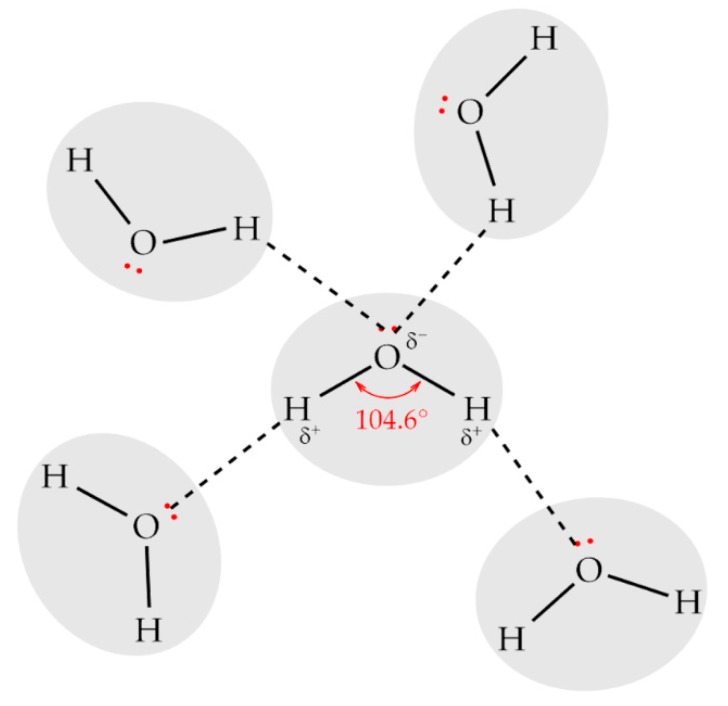
Structure of a 4-coordinated water molecule. Hydrogen atoms are positively charged (δ+) and oxygen atoms are negatively charged (δ−), which also present a lone pair of electrons. Solid lines indicate covalent bonds and dashed lines, hydrogen bonds.

Water is a small molecule composed of two hydrogen atoms covalently bonded to one oxygen atom. These bonds form a V-shaped structure with an angle of 104.6° (Figure 1) due to the two pairs of unused electrons of each oxygen atom, which tend to position as far from each other as they can to minimize the repulsion. The presence of these electrons causes a large dipole moment (i.e., uneven distribution of charges in the molecule), where the oxygen atom is slightly negatively charged, and the hydrogen atoms are slightly positively charged. This large dipole moment leads to a strong intermolecular interaction called hydrogen bonding, which is much weaker than the intramolecular covalent OH bond (23.3 kJ·mol^−1^ vs. 492 kJ·mol^−1^) [3]. The most predominant form is a 4-coordinated water molecule which forms hydrogen bonds with four different water molecules, i.e., each hydrogen atom pairs up with an oxygen atom of different water molecules, while the oxygen atoms pair up with two hydrogen atoms of different water molecules [4].

The behavior of pure water can be described by different models, which can be used to explain the peculiarities of water and its properties. In general, these models can be classified into two groups: (i) mixture or multicomponent models, where two or more water groups are present; and, (ii) continuum or uniformist models, in which each water molecule is influenced by the same intermolecular force. The flickering cluster model corresponds to a mixture model that suggests that clusters of hydrogen-bonded water are rapidly formed and swim in a medium of monomeric water molecules. However, this model fails to explain many of the water properties, as well as the nature of the clusters. On the other hand, the continuum model is a widely accepted model for water structure and explains water in terms of intact hydrogen bonds unlike the mixture model, where the hydrogen bonds are broken between the water molecules [2,5].

### 1.2. Water in Biological Systems

Water is very important in all biological systems. It is the major component of the body, at around 57% of the human weight. Without water, biochemical reactions would not be possible, as well as many other biological activities. For example, water is part of the functioning of enzymes when a substrate binds to the active site displacing water molecules [2]. When medical devices are implanted in the body, the material is in contact with water before any other biological component, such as proteins or cells [6]. Thus, it is reasonable to think that water plays a major role in material–body interactions in medical devices, specially mediating the biocompatibility of these materials [2].

### 1.3. Surface Water

The water network can be disturbed by external elements such as ions, solutes, and surfaces. When these elements become in contact with water, they reorganize its structure in order to minimize the energy of the system. In the case of surfaces, their interaction with water depends on properties such as structure, polarity, hydrophilicity or hydrophobicity, and composition of the surface. Water molecules reorder according to these factors, forming different conformations or layers at the vicinity of the surface. The influence of hydrophilic and hydrophobic surfaces on water structure has been widely studied as seen in the study by Li and collaborators, where the microscopic behavior of water on different surfaces of self-assembled monolayers was studied [7]. They suggested that water molecules neighboring a hydrophilic surface are more rigid and have better ordering than pure water. In contrast, when faced with a hydrophobic surface, water molecules have almost the same mobility and distribution as of pure water.

In particular, on hydrated polymers, different water “types” or “states” have been observed. These can be classified depending on the mobility of the water sorbed to the polymeric surface or its freezing temperature. Generally, there are three states of water, one with very low mobility and no freezing (even at −100 °C); one with slightly higher mobility and freezing temperature below 0 °C; and one with similar mobility and freezing temperature as bulk water. Further details about their characteristics and multiple denominations are given in the next section.

### 1.4. Biocompatibility

The concept of biocompatibility has been analyzed and defined thoroughly in order to comprise most of the factors that are implied in the material–body interaction. The most common definition is: “the ability of a material to perform with an appropriate host response in a specific application” [8]. This definition, although very generic, includes some essential characteristics that are expected for a material to have: (i) the material in the body must have a specified function and therefore it has to perform it; (ii) the host response that the material provokes has to be acceptable; and (iii) the response to a specific material will vary in different situations because the performance of the material depends on a specific application, in a specific tissue [9,10]. Given that biocompatibility changes according to the situation, it should not be considered as a property of a biomaterial, but a characteristic of a material-tissue interaction [10].

There are several factors that affect biocompatibility. Some of them are: composition of the material, production processes, structure (e.g., woven, knitted, polished, film, tube, sponge, sphere), surface characteristics (e.g., smoothness, porosity, rheology), surface treatments/coatings, location of the device, and expected host response on the interface (e.g., macrophage activation, biofilm formation, encapsulation, platelet adhesion) [10].

Taking these factors into account a more detailed definition of biocompatibility was proposed by Williams [10]:

“Biocompatibility refers to the ability of a biomaterial to perform its desired function with respect to a medical therapy, without eliciting any undesirable local or systemic effects in the recipient or beneficiary of that therapy, but generating the most beneficial cellular or tissue response in that specific situation, and optimizing the clinically relevant performance of that therapy.”

Biocompatibility evaluation can be performed by analyzing different biological effects that the material has in the body. The assessment of biocompatibility according to the ISO 10993 [11] is based on the material characteristics and its potential risk in the body under the conditions it is used. The biocompatibility test categories presented by the ISO 10993 are: cytotoxicity, hemocompatibility, degradation, implantation, sensitization, irritation or intracutaneous reactivity, acute system toxicity, material-mediated pyrogenicity, subacute or subchronic toxicity, genotoxicity, chronic toxicity, carcinogenicity, and reproductive or developmental toxicity. A brief description of the most relevant aspects of biocompatibility for this review is presented below:

- Citotoxicity: cell damage caused by direct contact with the material or by leached compounds.
- Hemocompatibility: effect on blood or blood components such as breakdown of blood cells, immunologic response, and thrombus formation.
- Degradation: breakdown of the device, which products might cause toxicity.
- Implantation: local effects of the implant on tissue.

In this review, hemocompatibility is evaluated by the adsorption of plasma proteins and platelet adhesion. It is known that when an implant makes contact with blood, plasma proteins adsorb to the surface. Fibrinogen (*F*g), albumin, and immunoglobulins are the most abundant, and along with fibronectin, vitronectin and the von Willebrand factor, these proteins are mediators of the platelet adhesion and thrombosis around the biomaterial [12]. In particular, fibrinogen, the main protein in blood plasma, is fundamental in blood coagulation and thrombosis induced by biomaterials. Activation of fibrinogen leads to platelet immobilization, activation, and aggregation, although the availability of platelet-binding sites is more important than the total amount of adsorbed fibrinogen in mediating platelet adhesion [12]. Either way, high fibrinogen adsorption and high platelet adhesion imply that the biomaterial is not inert in blood, hence it has low hemocompatibility.

There are few studies that consider water as a relevant factor on biocompatibility, and even fewer relate its states on the surface with its biocompatibility. This review highlights the role water and its states have on biocompatibility, specifically on the hemocompatibility of polymer surfaces.

## 2. Types of Water

### 2.1. Denominations

The study of water in different surfaces has been driven by the interest in investigating the effects of water on the functionality of these surfaces. As mentioned above, it includes hydrophobic/hydrophilic surfaces, metals, polymers, among others. Because of the large number of studies on this matter, a summary of the different denominations that water receives on surfaces is presented. 

Generally, water is classified in three states according to mobility or freezing temperature criteria. Early studies using nuclear magnetic resonance (NMR) suggested the possibility of an ordered water structure in the vicinity of different surfaces, called *bound* or *ice-like* water due to its low molecular mobility. Later, other types of water were identified: one with higher mobility than the so-called *bound* water, and another that behaves similarly to *bulk* or pure water. 

In most cases, the naming of the types of water has been based on its thermal behavior (i.e., crystallization), or on the mobility of their molecules (Table 1). Water can be classified according to low, intermediate, and high mobility at atmospheric conditions, and based on this criterion Hechter and collaborators [13], Sterling and Masuzawa [14], and McBrierty and collaborators [15] arrived at similar classification of the states of water with similar names. These three classifications were based on the NMR spectra of water, which are described in the next section. 

The other criterion used is the freezing temperature of water and the most common used denominations are the one presented by Higuchi and Iijima [16], Hatakeyama and Hatakeyama [17], and Tanaka and collaborators [18]. *Non-freezing* water does not freeze (even at −100 °C); *freezing bound* water freezes at temperatures below 0 °C; and *free* water freezes at 0 °C. Hirata and collaborators [19] described hydration water as the one that freezes below 0 °C or does not freeze at all. Therefore, hydration water is equivalent to *non-freezing* and *freezing bound* water, while *free* water is the same in both cases. Additionally, the mobility and the freezing temperature criteria can be considered equivalent, because more thermal energy in molecules translates in faster movement of them and thus molecular mobility increases with temperature. A third criterion was proposed by Aizawa and collaborators [20], and Jhon and Andrade [5] which was based on the thermal expansion of water molecules and the transition temperature of this property, i.e., the temperature at which thermal expansion changes.

**Figure 2:**
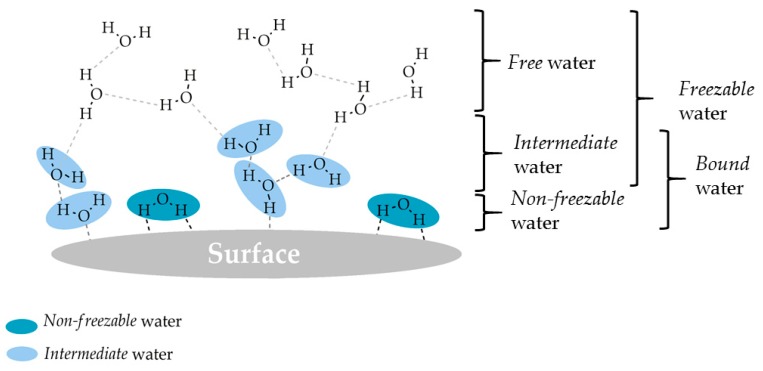
Types of water on surfaces with the denominations, their structure and interactions on surfaces.

In this review, we refer to the different types of water as: *non-freezable* water, *intermediate* water and *free* water, according to the freezing temperature criterion. *Non-freezable* and *intermediate* water are *bound* water, while *intermediate* and *free* water are *freezable* water (Figure 2). In terms of structure and interaction with surfaces, the three water types are characterized by the following [17,21]:

- *Non-freezable* water is tightly bound to the surface and the water-surface interactions are very strong, while water–water interactions are very weak.
- *Intermediate* water interacts moderately with the surface (stronger than *free* but weaker than *non-freezable* water), involving both water-surface and water–water interactions.
- *Free* water hardly interacts with the surface and there is mainly water–water interaction.

### 2.2. Water Measurement Techniques

Water content and water uptake are two denominations for total water sorbed in a sample. These can be can be measured by thermogravimetrical analysis (TGA), and their difference depends on whether the dry or wet sample is considered. Then, water uptake (WU) and water content (WC) can be calculated as follows:

(1) $$WU=\;\frac{w_{water}}{w_{dry}}=\frac{w_{wet}-w_{dry}}{w_{dry}}$$

(2) $$WC=\;\frac{w_{water}}{w_{wet}}=\frac{w_{wet}-w_{dry}}{w_{wet}}$$

where $w_{water}$, $w_{wet}$ and $w_{dry}$, correspond to the weight of sorbed water, wet sample and dry sample, respectively. 

There are several analytical methods to identify and/or quantify the different types of water on a surface. The three main techniques used are differential scanning calorimetry (DSC), NMR, and infrared (IR) spectroscopy. These techniques are further described below.

#### 2.2.1. Differential Scanning Calorimetry

The identification of the states of water on a surface and their quantities can be analyzed using DSC. It is widely used because the identification of *intermediate* water is easy due to the clear peaks associated with the phase transition. It has been used by Hatekayama and Hatekayama [17] to study the state of water in several insoluble and soluble polymers, such as cellulose, polyhydroxysterene, hyaluronic acid, and poly(vinyl alcohol). Besides polymers, DSC can also be used to identify the states of water on metals [22], ceramics [23], and food [24,25].

**Figure 3:**
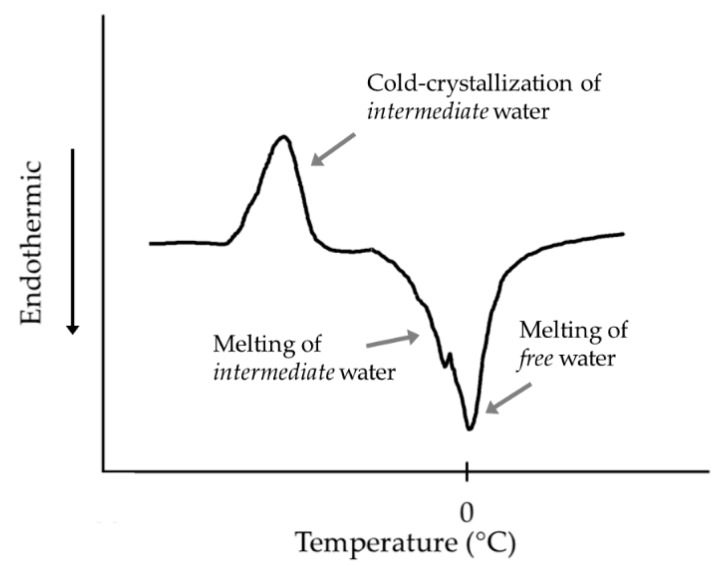
Scheme of a differential scanning calorimetry (DSC) thermogram of a hydrated surface with three states of water. *Non-freezable* water not visible in the thermograms. Modified from [26]. Reprinted by permission from Macmillan Publishers Ltd.: Polymer Journal 45: 701–710, copyright 2013.

DSC allows the observation of the phase transitions in a thermogram from which various information can be extracted: (i) changes in heat capacity, (ii) magnitude of the heat (exothermic or endothermic), (iii) shape of the exotherms or endotherms, and (iv) the temperature at which these phenomena occur [15]. Figure 3 shows a DSC thermogram of a hydrated surface containing the three types of water: *non-freezable*, *intermediate*, and *free* water. The exothermic peak below 0 °C corresponds to the cold-crystallization of water and indicates the presence of *intermediate* water. The endothermic peak at around 0 °C corresponds to the melting of *free* water and *intermediate* water where it corresponds.

The thermogram gives useful information to calculate the amount of the types of water. The mass of *intermediate* ($W_{int}$) and *free* water ($W_{f})$ can be calculated according to the following equations:

(3) Wint=Qcc∆Hcc

(4) Wf=Qm∆Hm−Wint

where $∆H_{cc}$ and $∆H_{m}$ are enthalpy changes in the cold-crystallization and the melting of ice, respectively; and, $Q_{cc}$ and $Q_{m}$ are the heat absorbed during the cold-crystallization process and the melting process, which are obtained from the area of the respective peaks in the thermogram. The enthalpy changes ($∆H_{cc}$ and $∆H_{m})$ are assumed to be the same as that of bulk water (334 J·g^−1^) [27].

The mass of *non-freezable* water ($W_{nf}$) is calculated as follows:

(5) $$W_{nf}=EWC\left(wt\%\right)-W_{int}-W_{f}$$

where EWC is the equilibrium water content of the sample.

The number of *non-freezable* ($N_{wnf}$) and *freezable* ($N_{wf}$) water molecules per polymer repeating unit can be calculated using the DSC information, as previously described [27,28]. The weight ratio ($WR_{nonfreezable}$) of *non-freezable* water/polymer, and the weight ratio ($WR_{freezable}$) of *freezable* water/polymer c be calculated using the following equations:

(6) $$WR_{nonfreezable}=\frac{w_{nonfreezable}}{w_{polymer}}=\frac{\left(EWC-w_{freezable}\right)}{w_{polymer}}$$

(7) $$WR_{freezable}=\frac{w_{freezable}}{w_{polymer}}$$

where $w_{nonfreezable}$, $w_{freezable},$ and $w_{polymer}$, are the weight percentages of *non-freezable* water, *freezable* water, and polymer, respectively. $w_{freezable}$ is obtained from the DSC thermograms and calculated as follows:

(8) $$w_{freezable}=\;\frac{Q_{m}}{∆H_{m}}\cdot 100\%$$

Finally, $N_{wnf}$ and $N_{wf}$ are obtained using the equations:

(9) $$N_{wnf}=\;\frac{M_{p}}{M_{water}}\cdot WR_{nonfreezable}$$

(10) $$N_{wf}=\;\frac{M_{p}}{M_{water}}\cdot WR_{freezable}$$

where $M_{p}$ is the molecular weight per polymer repeating unit, and $M_{water}$ is the molecular weight of water.

#### 2.2.2. Nuclear Magnetic Resonance 

NMR is used to measure the dynamic behavior of water, in contrast to the static state of water tmeasured by DSC. Most of the early investigations on water states used NMR for their identification, as in the case of Hechter and collaborators [13] with agar gels. It can also be used for other materials such as muscle tissue [29] and food, including vegetables [24], fruits [25], and dough [30].

**Figure 4:**
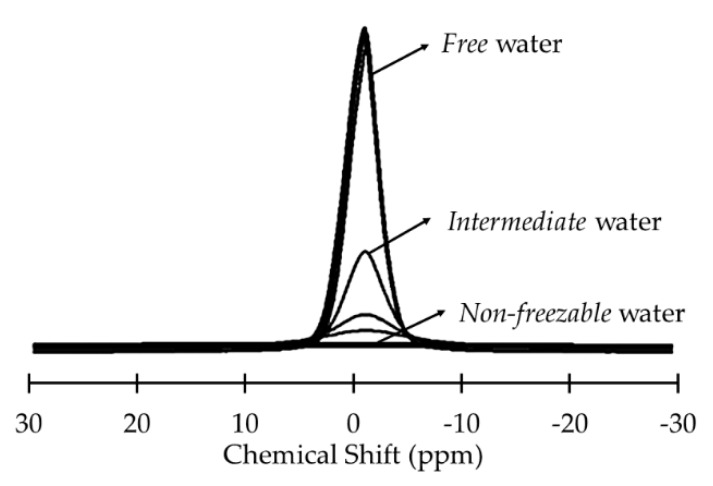
^2^H-NMR spectra of water on a surface at different temperatures. The narrower peaks correspond to temperatures above 0 °C, while broad lines correspond to temperatures below 0 °C. Modified from [32]. Copyright 2010 Taylor & Francis.

NMR is based on the line broadening observed when molecular mobility is interfered with. For *free* water, the resonance lines are narrow, but when water molecules interact with a solid, the mobility of the molecules may be impeded or nonexistent on the NMR time scale, resulting in broad resonance lines [31] (Figure 4). In general, a decrease in the signal intensity corresponds to an increased amount of *bound* water on the surface [32].

The NMR spectra of the different water states are as follows [32]:

- For *non-freezable* water the spectra is broad, showing low mobility due to strong interaction with the surface.
- For *free* water the spectra is narrow, very similar to *bulk* water, which means it has high mobility.
- For *intermediate* water the spectra is somewhere in between the spectra of the other two types of water, meaning that the mobility is intermediate.

The weight percentages of the different states of water can be calculated based on NMR data, though not many studies base their calculations on this technique. Sterling and Masuzawa [16] presented the methodology to determine them. From the NMR signal, height ($Peak_{H}$) of the peak and width ($Peak_{W}$) at half the height of the peak are measured. Then, the area of the peak is measured:

(11) $$A_{m}=Peak_{W}\cdot Peak_{H}$$

The width ($W_{w}$) and the area ($A_{w}$) of the peak of pure water are constant and considered to be 0.22 and 11.34, respectively. The calculated area ($A_{c}$) of the peak of the sample is based on the content of water in the sample:

(12) $$A_{c}=WC\cdot A_{w}$$

Then, the percentages of the water types can be calculated as follows:

(13) $$\%NF=100\cdot \left(1-\frac{A_{m}}{A_{c}}\right)$$

(14) %Int=100−(%NF+%Free)

(15) $$\%Free=100\cdot \left(\frac{H\cdot W_{w}}{A_{m}}\right)$$

where %NF, %Int and %Free correspond to the percentages of *non-freezable*, *intermediate*, and *free* water, respectively. 

#### 2.2.3. Fourier Transform Infrared Spectroscopy

Fourier transform infrared (FT-IR) spectroscopy is used to analyze the interaction of water molecules with functional groups of the surface. However, the information obtained cannot be used to calculate the amount of the different states of water. In general, IR analyzes the interaction of infrared light with a molecule by detecting the energy absorbance or transmittance of the molecule due to its vibration, which is known as the transmission sampling method. It is widely used to identify different molecules or molecular structure because functional groups vibrate at different energy levels [33]. The O–H stretching band region at around 3800–3000 cm^−1^ is of much interest because the interaction with water occurs through hydrogen bonding. Shifts of the wavenumber are associated with a change of the hydrogen bonding strength: the stronger the hydrogen bond, the lower the wavenumber shift [34]. The attenuated total reflection (ATR) is another sampling method of FT-IR, which is used to analyze thin and soft samples [26], as opposed to the transmission method which is used to analyze thin films and solids. With ATR-IR the infrared beam passes through a crystal, reflecting with the surface in contact with the sample positioned on top of it. The reflection produces an evanescent wave that goes through the sample. When the sample absorbs energy from the evanescent wave, this is attenuated, and the attenuated beam is analyzed to obtain the IR spectra. 

#### 2.2.4. Other Techniques

Other techniques have been used to study the types of water on surfaces. Raman spectroscopy can be used to analyze the structure and hydrogen bonding of sorbed water and is similar to IR. Raman spectroscopy detects changes in the polarizability of the molecular bonds, whereas IR detects changes in the dipole moment. The analysis extracted from these two are complementary and therefore provide a better insight of the water structure [35]. Recent research has been developed to clarify the origin of cold-crystallization of water combining wide-angle X-ray diffraction with DSC (XRD-DSC) [36]. The XRD determines the atomic and molecular structure of a crystal by the diffraction of incident X-rays, which with the combination of DSC allows identification of the origin of the phase transition.

## 3. Studies on the States of Water on Polymers and Their Effect on Biocompatibility

In this section, we analyze and discuss the impact of water states on the hemocompatibility, namely protein adsorption and/or platelet adhesion, of different polymers used for medical applications. In the first subsection, we include degradable polymers such as l-tyrosine derived polyarylates, poly(ethylene glycol) (PEG), a group of aliphatic carbonyls, and poly(lactic-*co*-glycolic acid) (PLGA). In the second subsection, some non-degradable polymers are analyzed, such as poly(meth)acrylates and poly(vinyl alcohol)s. 

### 3.1. Degradable Polymers

#### 3.1.1. l-Tyrosine Derived Polyarylates

**Figure 5:**
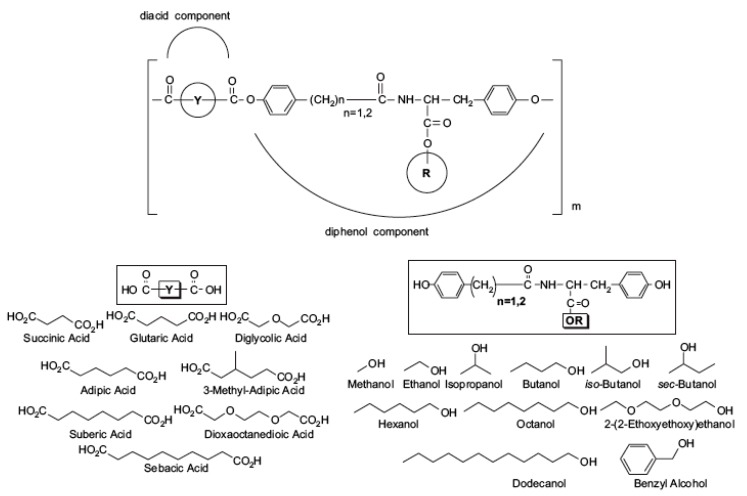
Structure of l-tyrosine derived polyarylates. Symbol Y represents diacids (lower **left**) and symbol R represents tyrosine-derived diphenols (lower **right**). The different compositions of R are: M = methyl, E = ethyl, B = butyl, H = hexyl, O = octyl, iP = isopropyl, sB = sec-butyl, Bn = benzyl. The number of methyl groups in the diphenol backbone is variable (*n* = 1 for HT, *n* = 2 for DT), where HT and DT stand for 4-hydroxyphenylacetic acid-tyrosine and desaminotyrosyl-tyrosine, respectively. Reproduced from [43] with permission of John Wiley & Sons.

l-Tyrosine-derived polyarylates are a family of 112 A-B-type copolymers with an alternating sequence of tyrosine-derived diphenol and an aliphatic diacid. They were first synthesized by Kohn and collaborators [37,38] (Figure 5). For this library, the number of oxygen or carbon atoms in the polymer backbone and pendent chain affects properties such as the glass transition temperature (*T*g) and the water contact angle. These polymers have been used to fabricate bone pins showing no significant inflammatory response in in vivo resorption studies [39]. Also, drug eluting implants near the eye area have been fabricated [40], as well as FDA approved devices for hernia repair and infection control in 2006 [41]. Recently, potential topical psoriasis therapy using drug loaded nanospheres have been developed which are based on amphiphilic block copolymers of PEG and l-tyrosine-derived polyarylate oligomers [42].

Previously, we investigated the influence of different properties of polymeric films on their WU behavior, due to the high variability of the latter reported in various studies [43]. From this study, the DSC curves for 21 of the used polymers were obtained, along with the WU data. From the former, the sample weight and the enthalpy change of the melting process of *freezable* water ($Q_{m}$) were extracted. Then, the WC and the number of *freezable* ($N_{wf}$) and *non-freezable* ($N_{wnf}$) water molecules per polymer repeating unit were calculated, as described in Section 2.2.1. Depending on the $N_{wf}$, polymers were classified into two groups: (i) low WC, from 0 to 0.31 and $N_{wf}$ less than 10, and (ii) high WC, from 0.45 to 0.6 and $N_{wf}$ more than 10.

**Figure 6:**
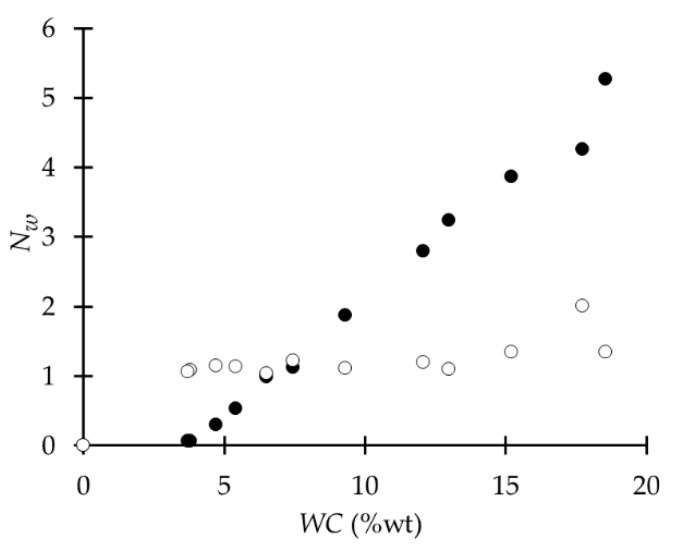
Example behavior of *non-freezable* water ($N_{wnf}$, o), and *freezable* water ($N_{wf}$, •) in relation to water content (WC) of poly(DTH adipate).

The analysis of the behavior of $N_{wnf}$ and $N_{wf}$ related to the WC of the polymer shows that the majority of them have similar behavior (Figure 6), where $N_{wnf}$ rises until a threshold from which it tends to remain constant, while $N_{wf}$ keeps rising as the polymer absorbs more water. However, when WC is very low (around 0.05) $N_{wnf}$ is higher than $N_{wf}$*.* This probably occurs because at lower WC the total amount of water is close to the amount of *non-freezable* water allowed in the polymer, and *freezable* water is in smaller or similar amounts.

**Figure 7:**
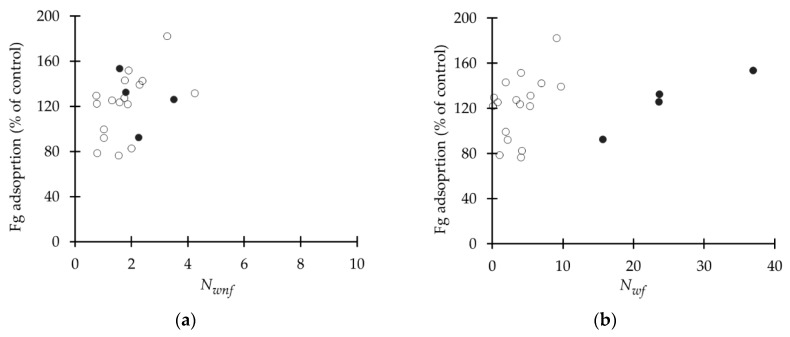
*F*g adsorption vs. $N_{w}$ in polyarylates (**a**): *non-freezable* water; (**b**): *freezable* water. (•) high WC, (o) low WC. X-axes are not in same scale.

Fibrinogen adsorption data was obtained from Weber and collaborators [44] (Table 2). Figure 7 shows the relation between $N_{w}$ and fibrinogen adsorption of *non-freezable* and *freezable* water. In the case of *non-freezable* water, there is no apparent relationship between the fibrinogen adsorbed to the surface, but for the low WC group there is a tendency to increase as the amount of water is increased. On the other hand, when only *freezable* water is analyzed, the low WC group has the same tendency as the previous case, although the correlation is lower in the latter. Although only four data points are available, the high *WC* group shows a direct relation between *WC* and fibrinogen adsorption. More experiments are required to make a conclusive statement about this relationship.

Polyarylates with longer ester and diacid groups adsorb less fibrinogen than those with shorter ester and diacid groups [47]. We hypothesize that this phenomenon occurs because longer chains allow the polymer to fold upon itself forming more hydrogen bonding between functional groups of the chain and water, as occurs with PEG chains [48]. This interaction could cause the formation of *intermediate* water that decreases the adsorption of proteins. 

From the data analyzed it can be concluded that at higher WC values, higher *freezable* water content is responsible for the higher fibrinogen adsorption and low hemocompatibility of polyarylates. There is not enough available data to conclude anything regarding *intermediate* water and its relation to hemocompatibility because we were only able to calculate the amount of *freezable* water, namely *intermediate* and *free* water together. We hypothesized that longer chains promote the formation of *intermediate* water, which decreases fibrinogen adsorption. In order to confirm this, further studies on water structure in polyarylates through NMR and IR are necessary.

#### 3.1.2. Poly(ethylene glycol)

**Figure 8:**
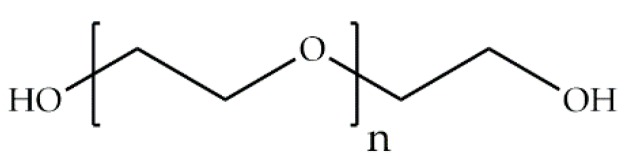
Molecular structure of poly(ethylene glycol) (PEG).

Poly(ethylene glycol) (PEG) is formed by the polymerization of ethylene oxide and corresponds to a neutral polyether (Figure 8). This hydrophilic polymer is especially relevant in the biomedical field because of its nontoxicity in the human body. In aqueous solution, PEG presents high mobility with a large exclusion volume; it also has a great capacity for water uptake, which depends on its molecular weight (*M_w_*). This polymer has been extensively used, as a blend or as a copolymer, to improve the biocompatibility and water solubility of other polymers with little chemical modification [49]. In tissue engineering, PEG has been used to form hydrogels, mostly in diblock, triblock, and multiblock copolymers with poly(lactic-*co*-glycolic acid) (PLGA) to improve the degradability of the PEG hydrogels [50]. In drug delivery systems, PEG is used as a stabilizer by coupling it to proteins, polypeptides, DNA, RNA, among others [51]. PEG has also been utilized as a coating for biosensors, especially those applied for blood glucose sampling and monitoring for their anti-fouling properties [52,53]. Some relevant PEGylated products include those for severe combined immunodeficiency and (Adagen^®^, Gaithersburg, MD, USA) acute lymphoblastic leukemia (Oncospar^®^, St. Helier, Jersey) [54]. Recent PEGylated products include therapy for age-related macular degeneration (Macugen^®^, New York City, NY, USA) and for Crohn’s disease and rheumatoid arthritis (Cimzia^®^, Smyrna, GA, USA) [51].

**Figure 9:**
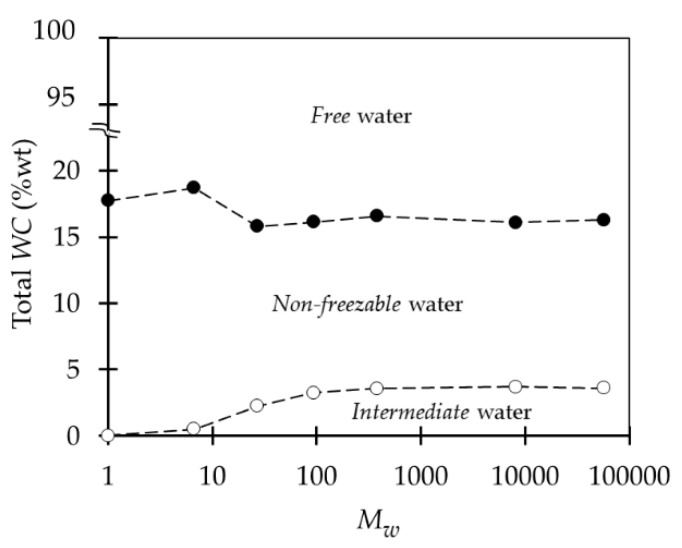
Water states distribution in PEG at different *M_w_*. (•) *Non-freezable* water; (o) *intermediate* water. Reprinted from [55] with permission of John Wiley & Sons.

The thermal behavior of aqueous PEG solutions with different *M_w_* was obtained with DSC measurements by Antonsen and Hoffman [48]. They explored and analyzed the effect of the *M_w_* on the amount of *bound* water and the low-temperature behavior of these solutions. They hypothesized that low *M_w_* PEG chains are only associated with *non-freezable* water and as the *M_w_* increases, the polymer chains fold upon themselves allowing *intermediate* water molecules to form. These results agree with the observations made by Yamauchi and Tamai [55] where at *M_w_* lower than 500, *intermediate* water was not detected, and then slowly increased with the *M_w_* until a constant amount of about 4% of total water was reached at around *M_w_* 2200 (Figure 9).

**Figure 10:**
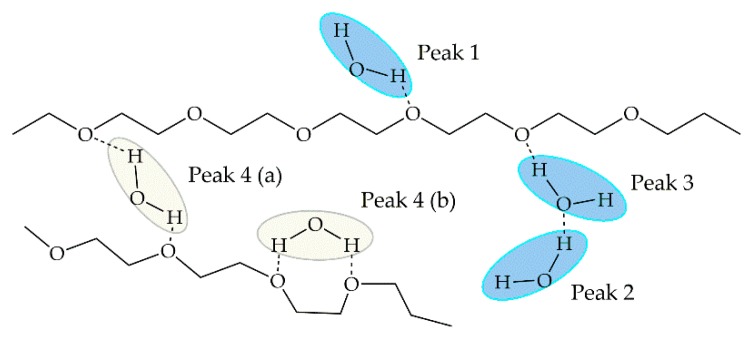
Proposed structure of water on hydrated PEG as shown in [56]. *Intermediate* water showed in blue; *non-freezable* water showed in yellow. Reprinted with permission from Langmuir 2001, 17, 1889–1895. Copyright 2001 American Chemical Society.

**Figure 11:**
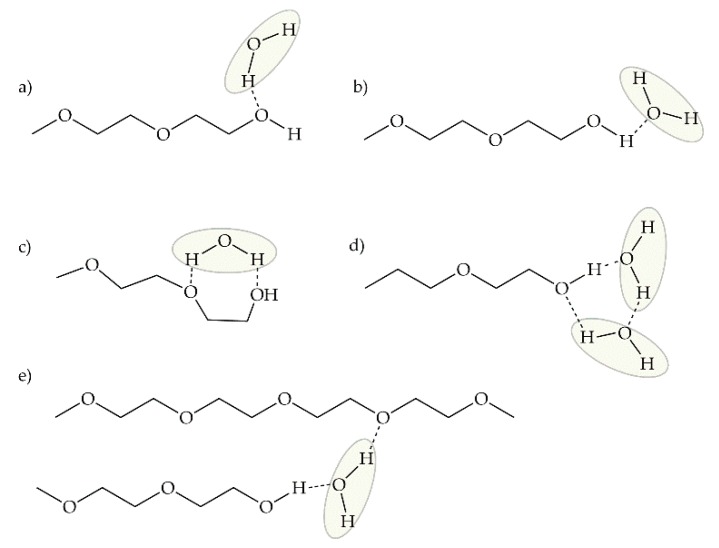
Proposed structure of water at the end of an hydrated PEG chain (peak X) as shown in [56]. Reprinted with permission from Kitano, H.; Ichikawa, K.; Ide, I.; Fukuda, M.; Mizuno, W. Fourier transform infrared study on the state of water sorbed to poly(ethylene glycol) films. Langmuir 2001, 17, 1889–1895. Copyright 2001 American Chemical Society.

Kitano and collaborators [56] conducted a FT-IR study to analyze the O-H stretching band peaks for sorbed water to PEG films of various *M_w_* and elucidate the effect of OH end groups of the polymer on the interaction with water molecules. In the IR spectra obtained (Figure 3 in [56]), different peaks of the O-H stretching band were observed, from which they proposed five types of sorbed water molecules: *binding* water, monomeric water binding to the ether oxygen atom of the polymer (peak 1); dimeric water, a water molecule which is hydrogen-bonded to another water molecule binding to PEG (peak 2) or a water molecule hydrogen-bonded to both a water molecule and the ether oxygen of PEG (peak 3); *bridging* water, association with two ether oxygen atoms (peak 4); and, hydrogen-bonded water to the end OH group (peak X). The latter was only observed in PEG films of low viscosity average molecular weight (*M_ν_* ≤ 28 K) due to the increase of OH ends of the polymer. Observing the proposed hydration structure of each water types (Figure 10 and Figure 11) and comparing to the three types of water, the following analogy can be drawn: peak 4 or *bridging* water corresponds to *non-freezable* water, while peaks 2 and 3 (dimeric water) correspond to *intermediate* water. Peak 1, although very similar to *bulk* water in the IR spectra, also corresponds to *intermediate* water given the higher mobility than bridging water. In the case of peak X, its IR spectra is very similar to peak 4, meaning water molecules are *tightly bound* to the polymer chain.

**Figure 12:**
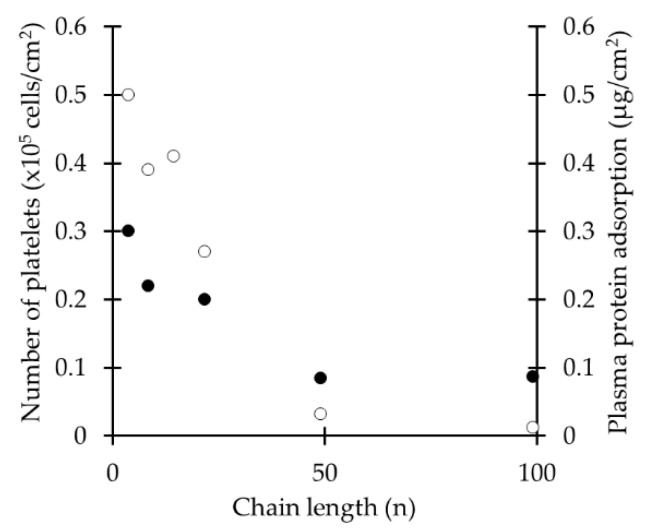
Effect of PEG chain length, immobilized to poly(methyl methacrylate) (PMMA) on platelet adhesion (•) and plasma protein adsorption (o). Data obtained from [57].

PEG presents good hemocompatibility. Both protein adsorption and platelet adhesion are a function of the *M_w_* below 2000 [48]. At low *M*w protein adsorption and platelet adhesion are high [57,58] because the amount of *intermediate* water is negligible. As described above, the increase of *M_w_* causes the formation of *intermediate* water in the polymer matrix until it reaches a maximum and stays constant at higher *M*_w_. At high *M*_w_, the folding of the chain and the subsequent formation of *intermediate* water molecules prevents adhesion to the polymer and to *non-freezable* water of high molecular-weight molecules like plasma proteins [48], avoiding the activation of these and hence, providing good hemocompatibility to the polymer. As shown in Figure 12, the longer the chain length of PEG immobilized to poly(methyl methacrylate) (PMMA), the lower the amount of adsorbed proteins and adhered platelets [57].

#### 3.1.3. Aliphatic Carbonyls

Aliphatic carbonyl polymers have ester or carbonate linkages facilitating the breakdown of their monomers and thus their degradation. This type of polymer has been broadly studied and applied in the biomedical field for the development of drug delivery systems and tissue engineering. Poly(dioxanone) (PDO) is mostly used for the manufacture of absorbable sutures [59] and has been recently studied in the development of degradable intravascular stents [60] and nanofibrous scaffolds [61]. Poly(ε-caprolactone) (PCL) and poly(trimethylene carbonate) (PTMC) are used in tissue engineering scaffolds and sutures [59]. These polymers have high biocompatibility, however there is little research available on the relation of the states of water on these polymers and their compatibility.

**Figure 13:**
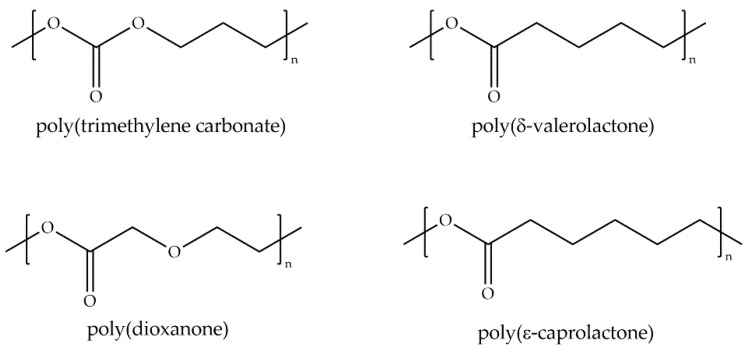
Molecular structure of aliphatic carbonyl polymers.

Tanaka and collaborators [62] studied PDO, PCL and PTMC (see Nomenclature Section) in addition to poly(δ-valerolactone) (PVL) (Figure 13) in order to elucidate the differences in their backbone structure on hydration and hemocompatibility. They found that a higher amount of *intermediate* water in PDO is related to its good hemocompatibility and the presence of ether bonds in the main chain of PDO are involved in the hydration and formation of *intermediate* water.

**Figure 14:**
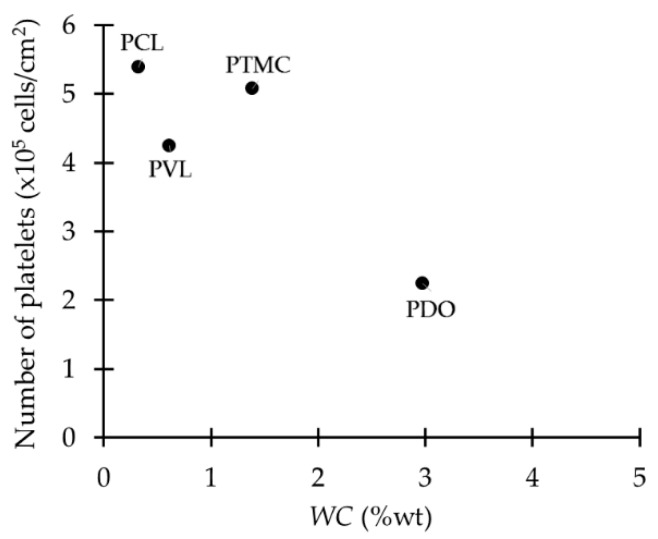
Relation of *intermediate* water content and platelet adhesion in aliphatic carbonyl polymers. Data extracted from [62].

The available data of equilibrium water content (EWC) and amount of types of water of PDO, PCL, PTMC and PVL were related to the amount of adhered platelets. In general terms, no relation between the total WU, *non-freezable* water and *free* water with platelet adhesion was found. However, there is a tendency of lower platelet adhesion when intermediate water is higher in quantity (Figure 14). 

**Figure 15:**
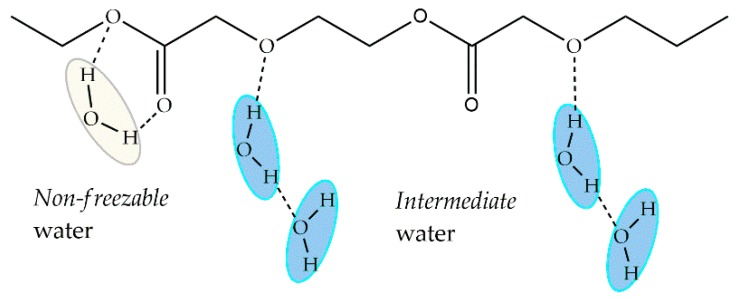
Proposed structure of *non-freezable* (yellow) and *intermediate* (blue) water sorbed on poly(dioxanone) (PDO).

The number of adhered platelets decreases considerably when *intermediate* water content is about 3 wt %, which occurs for PDO. The difference between PDO and the rest of the polymers is the ether bond in its backbone and it is believed that this bond contributes to the formation of hydrogen bonds and the increased amount of *intermediate* water, improving its hemocompatibility (Figure 15). At first glance at Figure 14, one could conclude that there is an inverse relationship between the number of platelets and the amount of *intermediate* water. However, the actual amount of *intermediate* water on PTMC might be overestimated because the author hypothesized that this water is not in the hydration layer formed by the polymeric chains and water, but spreads over this layer [62]. Therefore, the data of PTMC in Figure 14 should be closer to the Y-axis next to PCL and PVL, and the inverse relation is not conclusive anymore.

#### 3.1.4. Poly(lactic-*co*-glycolic) Acid

**Figure 16:**
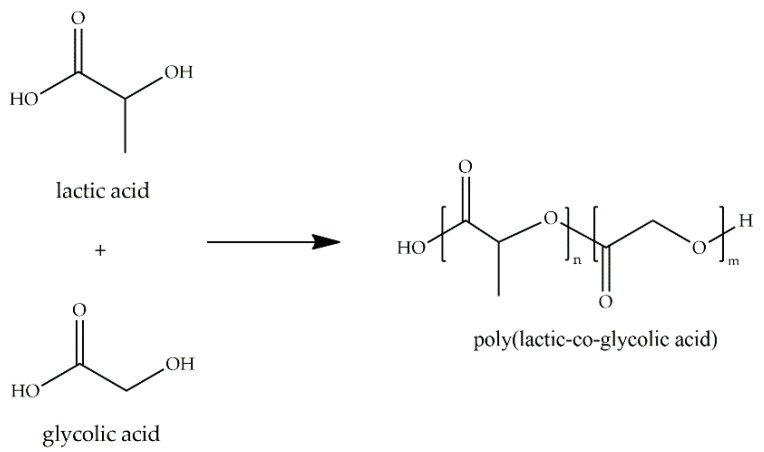
Molecular structure of poly(lactic-*co*-glycolic acid) (PLGA) and its monomers.

PLGA is the result of the copolymerization of lactic acid (LA) and glycolic acid (GA) and corresponds to a saturated poly(α-hydroxy ester) (Figure 16). This polymer presents good biocompatibility and biodegradability, which can be tailored by controlling the *M_w_* of the polymer and the ratio between lactic and glycolic acid [63]. Commonly used LA:GA ratios are 25:75, 50:50, 75:25, and 85:15, all of them with different *T*g values, degree of crystallinity, and degradation rates. Due to the wide range of properties this copolymer can have, it is one of the most popular in the pharmaceutical and medical industry. Many PLGA-based products have been approved by the FDA, such as sinus implants for the treatment of chronic rhinosinusitis [64], and injectable suspensions containing PLGA microspheres for the treatment of patients with acromegaly [65].

Blasi and collaborators [66] studied the effect of water on the *T*g of PLGA 50:50 in the early stage of hydration and the physical state of water within the hydrated polymer. They characterized the thermal behavior of the hydrated polymer using modulated DSC (MTDSC), a technique that allows the application of an oscillatory heating (or cooling) profile with improved resolution and enhanced sensitivity in comparison to conventional DSC [67]. From the cooling ramp of the MTDSC only one exothermic peak appears at around −20 °C, indicating the crystallization of water. The absence of a peak at 0 °C indicates that the peak at −20 °C does not correspond to cold-crystallization of *intermediate* water, but to supercooling of *free* water, thus no *intermediate* water is present in PLGA 50:50.

**Figure 17:**
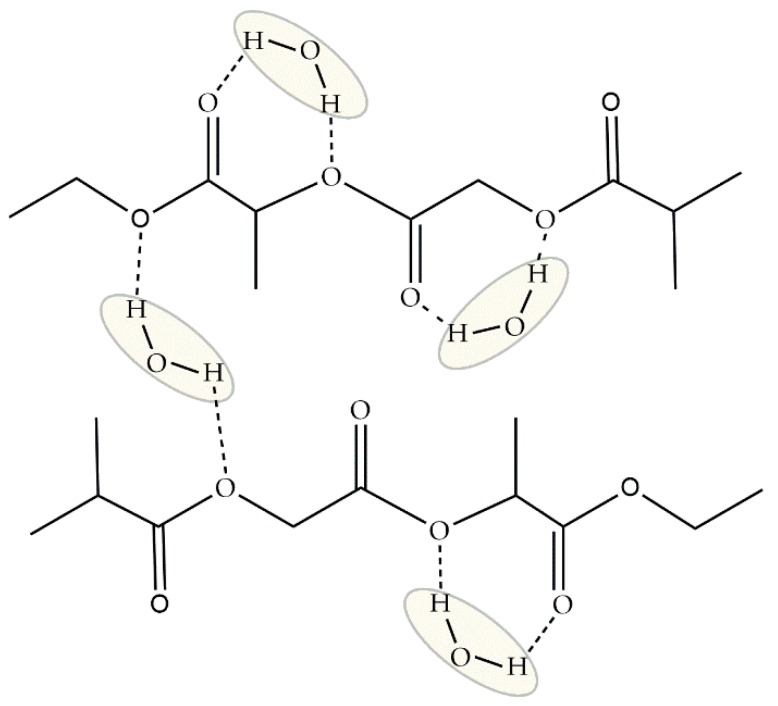
Proposed structure of *non-freezable* water sorbed on PLGA.

In terms of water structure, Blasi and collaborators [66] suggested that water molecules could directly interact with the polymer chain through hydrogen bonds with its hydrophilic groups (C=O and COO–). However, *intermediate* water was not identified, probably because of a lack of binding sites. *Intermediate* water, as studied in other polymers, can form either by binding weakly with a hydrophilic group or with another water molecule directly bound to the polymer. In this case, we suggest that *non-freezable* water simultaneously binds with two hydrophilic groups, as shown in Figure 17. Therefore, the remaining water molecules can only bind with one other, forming *free* water.

**Figure 18:**
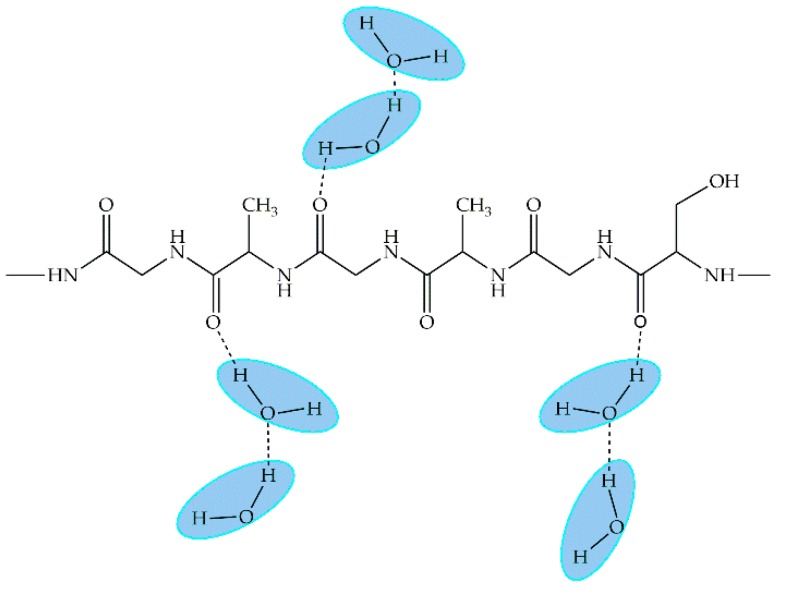
Proposed structure of *intermediate* water sorbed on silk fibroin.

The hemocompatibility of PLGA 50:50 is very low with an increased fibrinogen adsorption with respect to noncoated polypropylene (about 150%) [44]. Moreover, Liu and collaborators compared PLGA 85:15 and PLGA 85:15 immobilized with silk fibroin and demonstrated that the platelet adhesion, activation and the thrombogenic ability of the polymer by itself was higher than that of the treated one [68]. For example, untreated PLGA adhered over 550 × 10^5^ platelets/cm^2^ after 2 h incubation, while treated PLGA adhered only about 50 × 10^5^ platelets/cm^2^. Other biocompatibility aspects studied by them include vascular endothelial cell attachment and morphology, cell viability, transcription level of genes, and expression of proteins. When silk fibroin is immobilized to the polymer [68] an additional hydrophilic group is available for water molecules to bind. Then, *intermediate* water would form due to the presence of the carbonyl group of the fibroin (Figure 18), which would explain the improved hemocompatiblity and other biocompatibility aspects of treated PLGA. More studies on the water states in modified PLGA polymers are necessary to clarify if this improvement is due to the presence of *intermediate* water.

#### 3.1.5. Poly(vinyl alcohol)

**Figure 19:**
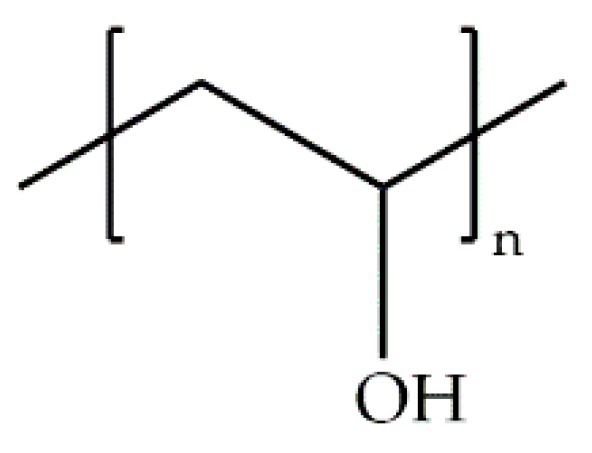
Molecular structure of poly(vinyl alcohol) (PVA).

Poly(vinyl alcohol) (PVA) is one of the few vinyl polymers that are water soluble and biodegradable (Figure 19). This polymer is used in many industries such as plastic, textile, paper, food, biomedical, and pharmaceutical. In the biomedical field, studies on the use of PVA for contact lenses, skin and artificial meniscus have been developed [69], while in the pharmaceutical industry, it is mainly used for drug delivery systems and many PVA-based products are currently available. Most of these products are in the form of tablets, but there are also transdermal/topical forms, and ophthalmic and implantable devices [69].

**Figure 20:**
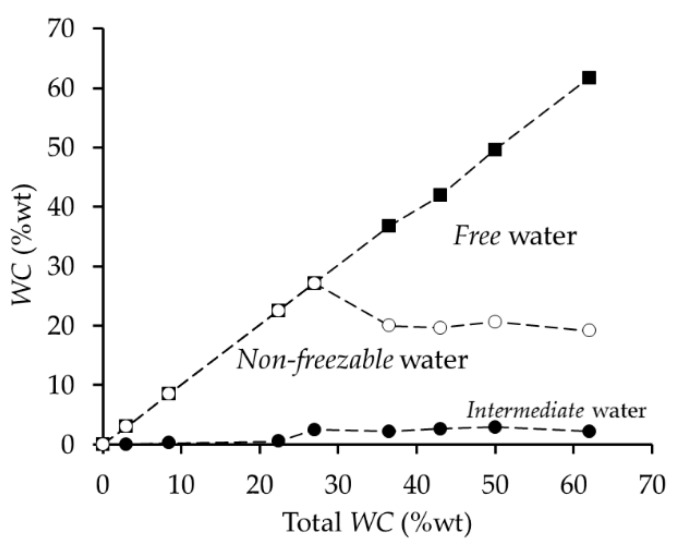
Water states distribution in PVA at different WC. (•) *Intermediate* water; (o) *non-freezable* water; (▪) *free* water. Data obtained from [70].

Hodge and collaborators [70] studied the states of water by DSC in PVA films. They observed that all the water sorbed into the polymer inhabits the amorphous region but also destroys crystalline regions. Therefore, as the polymer sorbs more water the crystalline region decreases. They also calculated the amount of each water state at different water content. When the total water content is below 22%, all of this corresponds to *non-freezable* water. Above this value, *intermediate* water appears and reaches a maximum of 2.5% while *free* water also appears increasing linearly until its saturation level of about 60% of total water (Figure 20).

The hemocompatibility of PVA is considered good and the adhered platelets inactive on the polymer surface [71]. This polymer PVA is an attractive polymer for tissue engineering applications due to its mechanical properties.; however, many studies have modified PVA by the addition of other polymers such as gelatin or dextran [69,72], in order to improve its hemocompatibility. When comparing PVA platelet adhesion (about 40 × 10^5^ platelets/cm^2^) [71] with other polymers presented in this review (e.g., PCL: about 5 × 10^5^ platelets/cm^2^), we can consider that platelet adhesion on PVA is high (i.e., low hemocompatibility). Taking into account the low *intermediate* water content in PVA films reported by Hodge and collaborators [70], where this value reached a maximum of 2.5%, we can conclude that low *intermediate* water content is related to high platelet adhesion, although it is not activated by contact with the polymeric surface.

### 3.2. Non-Degradable Polymers

#### 3.2.1. Poly(meth)acrylates

Polymethacrylates correspond to copolymers of methacrylic acid and acrylates. Copolymerization in different ratios provides a wide range of products with different tensile strengths and elongations. In general, methacrylates have a higher tensile strength and lower elongation than their corresponding acrylate [73]. In the medical field, polymethacrylates are used for the manufacture of contact lenses, artificial joints, dental implants, among others. The most studied polymer in these groups is PMMA and its applications include bone cement, intraocular lenses, and artificial kidney [74]. Eudragit^®^ (Essen, Germany) from Evonik is one of the commercial products of polymethacrylates and is used for functional solid oral dosage with specific drug delivery [75,76].

Tanaka and collaborators have carried out extensive research on the biocompatibility of poly(meth)acrylates, especially poly(2-methoxyethyl acrylate) (PMEA). They studied the structure of water in hydrated PMEA and compared it to water of poly(2-hydroxyethyl methacrylate) (PHEMA) and polyacrylates analogs [77]. They analyzed the phase transitions of water by DSC and performed a platelet adhesion test on the polymers. Their results show that PMEA shows a high hemocompatibility in comparison to the other polymers and this phenomenon appears to be dictated by the presence of *intermediate* water on the surface of the polymer and not by the total amount of water. 

**Figure 21:**
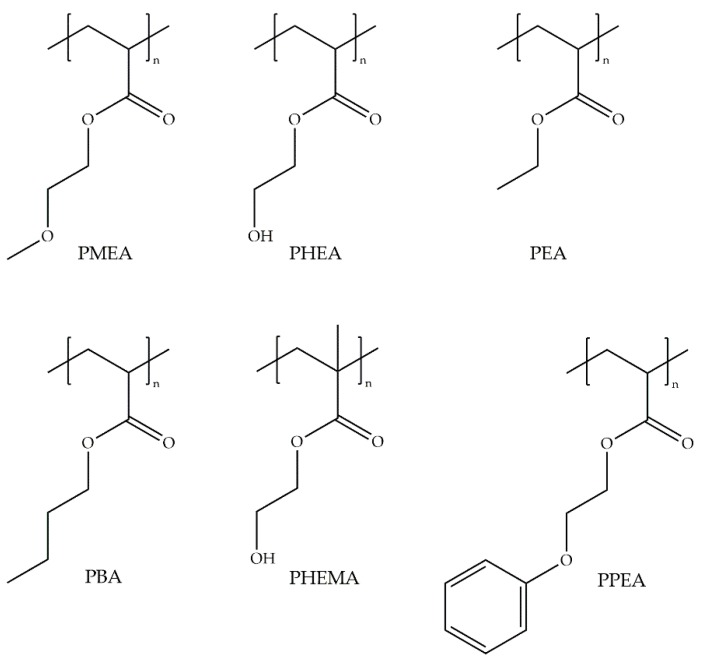
Molecular structure of poly(meth)acrylates.

**Figure 22:**
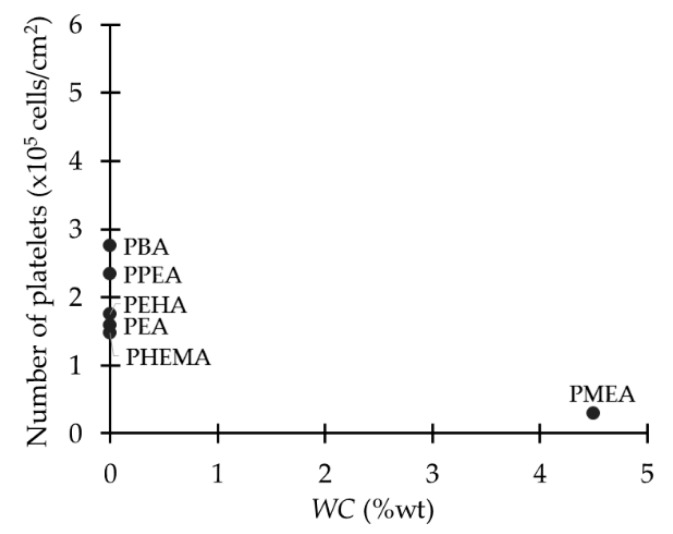
Relation of *intermediate* water content and platelet adhesion in poly(meth)acrylates. Data extracted from [77].

From this study, the available data of the different polymers were analyzed, namely: PMEA, PHEMA, poly(ethyl acrylate) (PEA), poly(2-phenoxyethyl acrylate) (PPEA), poly(2-ethylhexyl acrylate) (PEHA), and poly(n-butyl acrylate) (PBA) (Figure 21). EWC and amount of types of water were analyzed separately with the number of adhered platelets. In general terms, there is no relation between the total EWC, *non-freezable* water, and *free* water with platelet adhesion. However, as concluded by Tanaka and collaborators, the presence of *intermediate* water decreases the number of adhered platelets to the surface (Figure 22), hence the hemocompatibility is related to the presence of *intermediate* water.

The analysis of ^2^H-NMR and ^13^C-NMR of hydrated PMEA [32] shows that *non-freezable* water has a low mobility because it interacts strongly with the polymer chain, which stops this water freezing even below −100 °C. *Free* water has high mobility and it barely interacts with the polymer, resembling *bulk* water. From ATR-IR studies it was elucidated that *non-freezable* water interacts with the C=O groups in the PMEA side chain, while *free* water only interacts through hydrogen bonds with other water molecules and has no interaction with the polymer [34]. In the case of *intermediate* water, it interacts with the methoxy moiety (O–CH_3_) in the side chain terminal, both by hydrogen-bonding interaction. Although, this interaction is weaker than the one with *non-freezable* water, the ^2^H-NMR and ^13^C-NMR analysis show that *intermediate* water has intermediate mobility in comparison to the other types of water. It is hypothesized that because of these characteristics, the layer of *intermediate* water formed on the surface is more stable than *free* water and when it is sufficiently thick, it does not allow direct contact of cells or protein with the polymer surface or *non-freezable* water [26].

In general, *non-freezable* and *free* water are present in all surfaces of polymethacrylates. When fibrinogen approaches the surface through contact with *non-freezable* water it activates, it causes the adhesion of platelets to the polymer [26]. Due to the high mobility and its resemblance to bulk water, *free* water is not able to prevent this from happening. However, when *intermediate* water is present, it avoids the activation of plasma proteins, due to the moderate mobility of its molecules; and therefore, it plays a key role in their hemocompatibility.

#### 3.2.2. Poly(acrylonitrile)-*co*-*N*-2-vinyl-pyrrolidone

Polyacrylonitrile (PAN) is obtained from the polymerization of a vinyl group linked to a nitrile (acrylonitrile), and is mostly used as a copolymer in the production of plastics, as a precursor of carbon fiber and as separation membrane material in hemodialysis [78,79]. Some studies show the improvement of the biocompatibility of PAN by immobilizing other polymers such as PEG and *N*-vinyl-2-pyrrolidone (NVP) [80]. NVP is composed of a five-membered lactam (γ-Lactam) bound to a vinyl group; it has been used as a coating on cardiovascular devices showing improved biocompatibility [81]. Copolymers of acrylonitrile and NVP have been synthesized and applied in liver support systems [82] and as nanocarriers in drug delivery systems [83].

**Figure 23:**
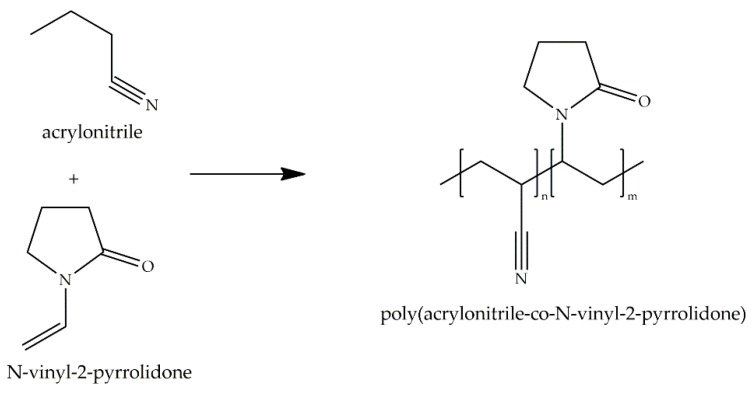
Molecular structure of poly(acrylonitrile)-*co*-*N*-2-vinyl-pyrrolidone (PANcNVP) and its monomers.

Wan and collaborators [79,84] compared the swelling behavior of PANcNVP films (Figure 23) with different NVP content: 7%, 15%, 22%, and 31%, with their hemocompatibility by platelet adhesion and plasma recalcification time (PRT) test. They observed that higher contents of NVP meant increased water uptake in the polymer, with increased amount of *non-freezable* and *freezable* water both determined by DSC and TGA (Table 3). They also conducted an FT-IR in transmission mode and in ATR to examine the diffusion and structure of water in the copolymers. From their calculations they identified three types of water in the surface of fully hydrated polymers (high NVP content): type III water (*free* water), with relatively weak hydrogen bonding with the nitrile group of PAN; type II water (*intermediate* water), monomeric or dimeric water molecules interacting with a carbonyl group in NVP; and, type I water (*non-freezable* water), bound to a carbonyl group and two or more water molecules. When analyzing the different hydrated copolymers, higher contents of NVP relate to higher amounts of *non-freezable* water, shown by the peak at wavenumbers below 3300 cm^−1^ in the FT-IR spectra. The presence of the carbonyl group of NVP allows the binding of more stable water molecules, namely, *non-freezable* water.

**Figure 24:**
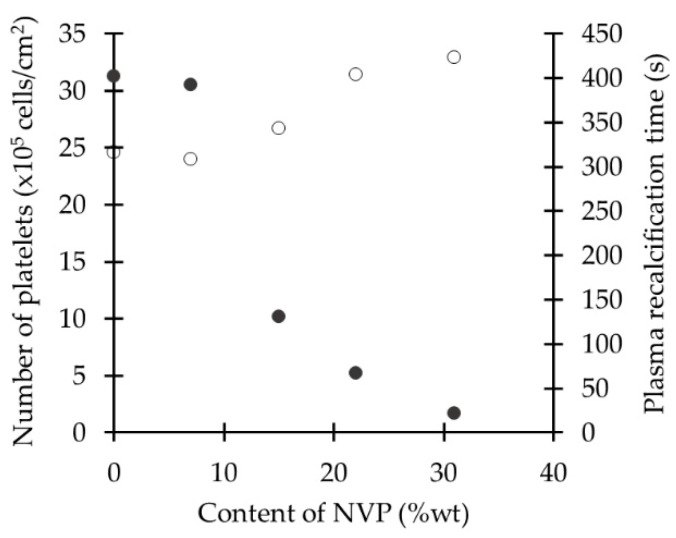
Relationship of *N*-vinyl-2-pyrrolidone (NVP) content with platelet adhesion (•) and plasma recalcification (PRT) time (o) on PANcNVP. Reprinted from [79] with permission of John Wiley & Sons.

Hemocompatibility tests showed that with higher amounts of NVP the number of platelets adhered to the surface was less than with samples with lower amounts of NVP, and these platelets conserved much of their original shape, indicating an inactivated state. The high content of NVP also increased the PRT, which indicates that coagulation around the polymer occurs more slowly than in copolymers with low NVP [79] (Figure 24). Relating this information with the water states data, the study suggests that the improved hemocompatibility is due to the higher amount of *non-freezable* water present in the polymer-water system. However, in the calculation of *non-freezable* and *freezable* water the authors did not differentiate the latter between *intermediate* and *free* water. They did however identify the three different states of water in the polymer-water system through IR analysis. Both *non-freezable* and *intermediate* water can be responsible for the improved hemocompatibility of the polymer and determining the amount of *intermediate* water with DSC or NMR will provide more information on its role on hemocompatibility.

## 4. Discussion

### 4.1. Water States in Polymers

Among the studies presented above, some of them analyzed the states of water as *non-freezable* and *freezable* water, understanding the latter as the sum of *intermediate* and *free* water. In the case of some polymethacrylates and PLGA, *intermediate* water was not identified. However, *non-freezable* water was always present in the polymer-water matrix analyzed. Also, based on the analysis in polyarylates, PEG and PVA, as the polymer hydrates, *non-freezable* water increases until it reaches a limit from which it remains constant at higher hydration levels. This occurs because as the polymer becomes more hydrated, there are not enough hydrogen bonding sites to form *non-freezable* water. 

The structure of the polymer determines the formation of the different types of water. Functional groups in the polymeric chain interact with different strength levels with water through hydrogen bonds. As seen in aliphatic carbonyls, the carbonyl group bonds to water forming *non-freezable* water but no *intermediate* water. The same happens in PANcNVP and the carbonyl group of the NVP. The ether group of PDO and PMEA binds to water and due to its moderate strength, *intermediate* water is formed. However, *intermediate* water can also form when a water molecule binds to another which is directly bound to the polymer. In this case, the indirectly bound water molecule has higher mobility than the directly bound one.

The length of the polymeric chains of the *M_w_* also affects the formation of the different states of water. When *M_w_* is higher, larger polymeric chains tend to fold upon themselves and functional groups of the chain are closer to each other. This induces the formation of *intermediate* water bridging these groups and supplying more stability to the folded polymer. Additionally, if the end group of a polymer has the capacity to form hydrogen bonds, then in low *M_w_* chains, where more end groups are available, more water molecules can bind to them and more *non-freezable* water is formed.

### 4.2. Biological Response in Polymers

**Figure 25:**
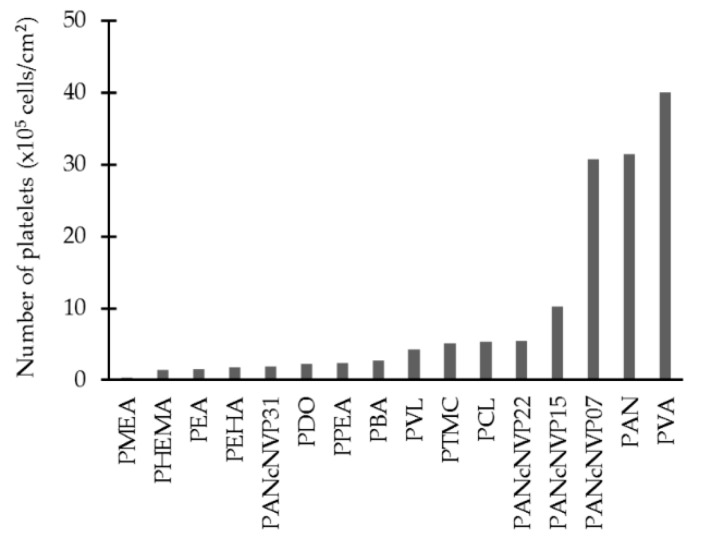
Platelet adhesion on different polymers.

Most of the biological response data presented here was about platelet adhesion. A polymer with a high amount of adhered platelets is considered to have low hemocompatibility as in the case of PLGA, which has about 550 × 10^5^ platelets/cm^2^ [68]. However, when comparing polymers with lower platelet adhesion, there is a lack of consensus on what is low, moderate or high hemocompatibility. Figure 25 shows the number of adhered platelets on different polymers. In the study of Tanaka and collaborators [77] they state that PMEA has excellent hemocompatibility while the other poly(meth)acrylates (PHEMA, PEA, PEHA, PPEA, and PBA) have low hemocompatibility. However, when compared to other polymers, like PAN or PVA, which are considered to have moderate hemocompatibility, poly(meth)acrylates have high hemocompatibility.

### 4.3. Effect of Water States on Biological Response

In most polymers studied, when *intermediate* water is present, low platelet adhesion is observed, which means high hemocompatibility. This relation is clear in PEG, poly(meth)acrylates, aliphatic carbonyls, and PLGA, although there is no mathematical relation between the content of *intermediate* water and the amount of adhered platelets. *Intermediate* water in the polymer is more stable and has higher mobility than *free* water, thus when it is sufficiently thick it prevents the activation of the protein by avoiding direct contact with *non-freezable* water. The main conclusions of each polymer are summarized in Table 4. 

Tanaka and collaborators [77] did not detect any *intermediate* water in poly(meth)acrylates except for PMEA. However, these polymers have high hemocompatibility in relation to other polymers such as PAN and therefore it would be expected that poly(meth)acrylates (PBA, PPEA, PEHA, PHEMA, and PEA) have some *intermediate* water of about 3 wt %. The apparent absence of *intermediate* water in these polymers could be confirmed by the absence of an exothermic peak of cold-crystallization in the heating DSC thermogram. However, the cooling thermogram shows the presence of *intermediate* water with a second peak below 0 °C, which the authors might not have considered. Additionally, in the heating thermograms a single peak with a shoulder on the lower temperature region is often considered as the effect of the melting of *intermediate* water, which is present in some heating thermograms of poly(meth)acrylates, therefore it cannot be definitely concluded that there is no presence of *intermediate* water. 

Considering PEG, aliphatic carbonyls and poly(meth)acrylates, a limit of about 3 wt % for *intermediate* water can be established, from which platelets adhere less to the surface and the polymer has higher hemocompatibility, i.e., at contents of *intermediate* water above 3 wt %, platelet adhesion is low, and below 3 wt %, platelet adhesion is high. In order to find the mathematic relationship between these two properties, *intermediate* water content should be calculated not only for the afore mentioned poly(meth)acrylates (where no *intermediate* water was detected), but also for polyarylates and PANcNVP.

For polymers such as PANcNVP and polyarylates, the available data is not sufficient to make similar conclusions, where the presence of *intermediate* water means low platelet adhesion. However, in polyarylates, at high water content, high *freezable* water relates directly to platelet adhesion. This *freezable* water corresponds to *intermediate* and *free* water, therefore considering the previous observation for poly(methyl methacrylates), it can be suggested that in polyarylates at high water content, higher *freezable* water contains less *intermediate* water, which would explain high platelet adhesion at high *freezable* water content. For PANcNVP, Wan and collaborators concluded that high content of *non-freezable* water means low platelet adhesion on the polymers. This conclusion was made after calculating the content of *non-freezable* and *freezable* water in the polymer and relating it to the content of NVP, and then to platelet adhesion. As in polyarylates, they calculated *intermediate* water and *free* water as *freezable* water; they also detected the presence of all three water types in the hydrated polymer. However, they did not calculate the content of these two states separately, in which case it could happen that at higher NVP content *intermediate* water is high, relating it to low platelet adhesion. 

In the case of PVA, the presence of *intermediate* water implies moderate platelet adhesion. This polymer has a maximum content of *intermediate* water of about 2.5 wt %, which is lower than the limit of 3 wt %, therefore hemocompatibility can be considered as low. In fact, data on platelet adhesion shows that PVA adheres about 40 × 10^5^ platelets/cm^2^, a higher value than the one reported for poly(meth)acrylates, PEG and aliphatic carbonyls, but lower than PLGA. Then, in this case, the relation is that low *intermediate* water content relates to high platelet adhesion. It is hypothesized that this relation is due to the low stability of *intermediate* water in this polymer, resembling *free* water, which is not enough to prevent the contact of proteins with *non-freezable* water. Another alternative is that *intermediate* water is clustered in the polymer matrix, leaving free regions of this type of water and allowing direct contact of the proteins. 

Finally, the improvement on biocompatibility of PLGA 85:15 by silk fibroin may be due to the formation of *intermediate* water. Although, the states of water in the polymer have not been identified nor calculated, it is hypothesized that the presence of the carbonyl group in silk fibroin allows *intermediate* water to form, improving the hemocompatibility. Hence, the presence of that water could also improve the other biocompatibility aspects of the scaffold, i.e., cell attachment and morphology, cell viability, transcription level of genes, and expression of proteins. This would imply that the presence of *intermediate* water not only could improves hemocompatibility of polymers, but also promotes other biocompatibility aspects. This preliminary conclusion should be demonstrated with further research on the states of water and their impact on biocompatibility.

## 5. Conclusions

Water and its states influence polymer hemocompatibility. In polymers such as PEG, aliphatic carbonyls, poly(meth)acrylates, PLGA, and PVA, *intermediate* water content of at least 3 wt % relates to low platelet adhesion and higher hemocompatibility than those with less or none, which is due to the better stability that this state of water has in comparison to *free* water, preventing platelets contacting *non-freezable* water and activating it. However, it is necessary to conduct further studies for the identification and measurement of *intermediate* water utilizing techniques like DSC and IR to support this conclusion. Furthermore, future challenges include the analysis of the relation of water states with other aspects of biocompatibility than hemocompatibility, and of the mechanism by which *non-freezable*, *intermediate*, and *free* water interact with biological components to provide more information on the role of water in biological response.

This review presents relevant information that will aid in the process of polymer selection and polymeric materials design given a certain application. Hydration states of water are not only relevant for polymers in the medical field but also for other materials and areas such as in the food industry, sewage treatment, among others. Water must not be considered as an inert solvent and, on the contrary, it must be noted that water does interact with solutes, forming different states, which interact with the material and are able to modulate their properties. Therefore, it is very important to consider water and its states in any process that has contact with water.

## Figures and Tables

**Table 1:** Denominations of the types of surface water based on different criteria.

Criteria	Types of Water		Reference
	Name	Criteria	
Rigidity and mobility	*Ice-like* water ^a^	Very low mobility	[13]
	*Intermediate* between ice-like and *free* water ^b^	Intermediate mobility	
	*Free* water ^c^	High mobility	
	*Solid* water ^a^ (*glass-like* or *ice-like*)	Very low mobility	[14]
	*Bound* water ^b^	Intermediate mobility	
	*Free* water ^c^ (very loosely bound or liquid)	High mobility	
	*Tightly bound* water ^a^	Very low mobility at temperatures <230 K	[15]
	*Loosely bound* water ^b^	Low mobility in the range 230–260 K	
	*Free* or *bulk-like* water ^c^	Mobility similar to bulk water at around 273 K	
Freezing temperature	*Hydration* water ^d^	Freezes sub-zero or does not freeze	[19]
	*Free* water ^e^	Freezes at 0 °C	
	*Bulk-like* water	Normal mobility as normal melting point is approached	
	*Non-freezing* water or *Non-freezing bound* water ^f^	No crystallization (no freezing)	[16,17,18]
	*Freezing-bound* water or *Intermediate* water ^g^	Crystallization under 0 °C	
	*Free* water ^e^	Normal crystallization of water	
Thermal expansion	*Hydrated* water	Transition temperature: Non at −30 to 0 °C	[5,20]
	*Interfacial* water	Transition temperature: −20 to 0 °C	
	“Normal” or *bulk* water	Transition temperature: 0 °C	

^a^ *Ice-like* water, *solid* water and *tightly bound* water are equivalent. ^b^ *Intermediate* between *ice-like* and *free* water, *bound* water and *loosely bound* water are equivalent. ^c^ *Free* water in the three references are equivalent. ^d^ Corresponds to ^f^ + ^g^. ^e^ Same *free* water by freezing temperature criterion.

**Table 2:** Data of each polyarylate measured and calculated from [45,46]. Fibrinogen (*F*g) adsorption data extracted from [44].

Polymer	$N_{wf}$	$N_{wnf}$	WC Max (wt %)	*F*g Adsorption (% of Control)	Group
poly(DTO succinate)	0.14	0.77	4	121.97	Low
poly(DTB succinate)	0.28	0.76	4	129.36	
poly(HTE adipate)	0.82	1.31	8	125.19	
poly(DTO adipate)	1.08	0.8	6	78.30	
poly(DTM adipate)	1.92	1.77	13	142.69	
poly(DTM sebacate)	1.98	1.03	12	99.14	
poly(DTH suberate)	2.25	1.03	10	91.68	
poly(DTB adipate)	3.45	1.76	19	127.12	
poly(DTB glutarate)	3.95	1.59	19	123.38	
poly(HTH adipate)	4.13	1.55	16	76.20	
poly(DTE glutarate)	4.13	1.91	22	151.44	
poly(DTH adipate)	4.26	2.01	18	82.27	
poly(DTiP adipate)	5.39	1.87	22	121.76	
poly(DTE adipate)	5.45	4.26	27	131.21	
poly(DTBn adipate)	6.98	2.40	30	142.16	
poly(HTE succinate)	9.14	3.27	30	182.15	
poly(DTBn methyl adipate	9.77	2.30	31	138.98	
poly(DTBn suberate)	15.71	2.26	47	92.10	High
poly(DTM (R)(+) methyl adipate)	23.67	3.51	56	125.70	
poly(DTsB glutarate)	23.70	1.80	49	132.32	
poly(DTsB (R)(+) methyladipate)	36.98	1.59	58	153.27	

**Table 3:** Water content of total, *non-freezable* and *freezable* water in PANcNVP. Extracted from [79].

Content of NVP (wt %)	Total Water (wt %)	*Non-Freezable* Water (wt %)	*Freezable* Water (wt %)
0	29.7	4.6	25.1
7	30.6	5.2	25.4
15	43.3	9.6	33.7
22	55.5	16.0	39.5
31	58.3	19.3	39.0

**Table 4:** Summary of principal observations and conclusions in each polymer.

Polymer	Types of Water Measured	Biological Response Measured	Observations	References	Conclusions
PEG	*Free*, *intermediate* and non-*freezable*		*Intermediate* water is negligible at low *M*w and increases with *M*w until a constant value	[48,55]	Presence of *intermediate* water means low protein adsorption and platelet adhesion
		Platelet adhesion & plasma protein adsorption	Low protein adsorption and platelet adhesion	[48,57]	
Aliphatic carbonyls	*Free*, *intermediate* and non-*freezable*	Platelet adhesion	There is lower platelet adhesion when *intermediate* water is present.	[62]	Presence of *intermediate* water means low platelet adhesion [62]
Poly(meth)acrylates	*Free*, *intermediate* and non-*freezable*	Platelet adhesion	*Intermediate* water present only in PMEA is responsible for its excellent hemocompatibility.	[18]	Presence of *intermediate* water means low platelet adhesion [18]
PLGA	*Free*, *intermediate* and non-*freezable*		No presence of *intermediate* water	[66]	Absence of *intermediate* water means high platelet adhesion.
		Fibrinogen adsorption & platelet adhesion	High fibrinogen adsorption and platelet adhesion	[44,68]	
		Cell attachment, morphology, viability; transcription level of genes and expression of proteins	PLGA with silk-fibroin has better biocompatibility	[68]	Presence of carbonyl group of fibroin allows *intermediate* water formation and better biocompatibility
PVA	*Free*, *intermediate* and non-*freezable*		PVA films have low *intermediate* water content	[70]	Low *intermediate* water content means high platelet adhesion (inactive state)
		Platelet adhesion	High platelet adhesion but in inactive state.	[71]	
PANcNVP	*Free*, *intermediate* and non-*freezable*		Three types of water present Higher NVP means higher *non-freezable* water content	[79] [84]	High content of *non-freezable* water means less platelet adhesion [84] *Intermediate* water could influence hemocompatibility (not enough data for conclusions)
		Platelet adhesion & PRT	Higher amounts of NVP led to less platelet adhesion and increase of PRT	[79]	
Polyarylates	*Freezable* and *non-freezable*		In polymers with WC over 10%, *non-freezable* water reaches a threshold lower than *freezable* water.	[43]	At high WC, high *freezable* water means high fibrinogen adsorption *Intermediate* water (implicit in *freezable* water) could influence hemocompatibility (not enough data for conclusions)
		Fibrinogen adsorption	Polymers with longer ester and diacid chains adsorb less fibrinogen	[44]	

## Nomenclature

∆H	Enthalpy change of melting of ice of *bulk* water
$∆H_{m}$	Enthalpy change of melting of ice
$∆H_{cc}$	Enthalpy change of the cold-crystallization of ice
%*Free*	Weight percentage of *free* water
%*Int*	Weight percentage of *intermediate* water
%*NF*	Weight percentage of *non-freezable* water
$A_{c}$	Calculated area of peak of NRM signal
$A_{m}$	Measured area of peak of NRM signal
$A_{w}\;$	Area of peak of NRM signal of pure water
ATR	Attenuated total reflection
*C_p_*	Specific heat capacity
DSC	Differential scanning calorimetry
DTB	Desaminotyrosyl-tyrosine butyl ester
DTBn	Desaminotyrosyl-tyrosine benzyl ester
DTE	Desaminotyrosyl-tyrosine ethyl ester
DTH	Desaminotyrosyl-tyrosine hexyl ester
DTiP	Desaminotyrosyl-tyrosine isopropyl ester
DTM	Desaminotyrosyl-tyrosine methyl ester
DTO	Desaminotyrosyl-tyrosine octyl ester
DTsb	Desaminotyrosyl-tyrosine sec-butyl ester
EWC	Equilibrium water content
*F*g	Fibrinogen
FT-IR	Fourier transform infrared
GA	Glycolic acid
HTE	4-hydroxyphenylacetic acid-tyrosine ethyl ester
HTH	4-hydroxyphenylacetic acid-tyrosine hexyl ester
IR	Infrared
LA	Lactic acid
$M_{p}$	Molecular weight per polymer repeating unit
MTDSC	Modulated differential scanning calorimetry
M_ν_	Viscosity-average molecular weight
$M_{water}$	Molecular weight of water
*M_w_*	Molecular weight
NMR	Nuclear magnetic resonance
NVP	*N*-vinyl-2-pyrrolidone
$N_{w}$	Number of water molecules per polymer repeating unit
$N_{wf}$	Number of *free* water molecules per polymer repeating unit
$N_{wnf}$	Number of *non-freezable* water molecules per polymer repeating unit
PAN	Poly(acrylonitrile)
PANcNVP	Poly(acrylonitrile)-*co*-*N*-2-vinyl-pyrrolidone
PBA	Poly(*n*-butyl acrylate)
PCL	Poly(ε-caprolactone)
PDO	Poly(dioxanone)
PEA	Poly(ethyl acrylate)
$Peak_{H}$	Height of peak in NMR signal
$Peak_{W}$	Width of the peak in NMR signal
PEG	Poly(ethylene glycol)
PEHA	Poly(2-ethylhexyl acrylate)
PHEMA	Poly(2-hydroxyethyl methacrylate)
PLGA	Poly(lactic-*co*-glycolic acid)
PMEA	Poly(2-methoxyethyl acrylate)
PMMA	Poly(methyl methacrylate)
PPEA	Poly(2-phenoxyethyl acrylate)
PRT	Plasma recalcification time
PTMC	Poly(trimethylene carbonate)
PVA	Poly(vinyl alcohol)
PVL	Poly(δ-valerolactone)
$Q_{cc}$	Heat associated to cold-crystallization process
$Q_{m}$	Heat associated to melting process
SEM	Scanning electron microscope
$T_{g}$	Glass transition temperature
TGA	Thermogravimetric analysis
WU	Water content
$w_{dry}$	Weight of dry sample
$W_{f}$	Mass of *free* water
$w_{freezable}$	Weight percentage of *freezable* water
$W_{int}$	Mass of *intermediate* water
$W_{nf}$	Mass of *non-freezable* water
$w_{nonfreezable}$	Weight percentage of *non-freezable* water
$w_{polymer}$	Weight percentage of polymer
$WR_{freezable}$	Weight ratio of *freezable* water:polymer
$WR_{freezable}$	Weight ratio of *non-freezable* water:polymer
WU	Water uptake
$w_{water}$	Weight of sorbed water
$w_{wet}$	Weight of wet sample
XRD-DSC	X-ray diffraction with DSC
